# Characterization of furathiocarb metabolism in in-vitro human liver microsomes and recombinant cytochrome P450 enzymes

**DOI:** 10.1016/j.toxrep.2022.03.046

**Published:** 2022-04-01

**Authors:** Khaled Abass, Petri Reponen, Walaa F. Alsanie, Arja Rautio, Olavi Pelkonen

**Affiliations:** a Arctic Health, Faculty of Medicine, University of Oulu, P.O. Box 7300, FI-90014, Finland; b Pharmacology and Toxicology Unit, Research Unit of Biomedicine, University of Oulu, P.O. Box 5000, Oulu FI-90014, Finland; c Department of Pesticides, Menoufia University, P.O. Box 32511, Egypt; d Department of Clinical Laboratory Sciences, The Faculty of Applied Medical Sciences & Centre of Biomedical Sciences Research (CBSR), Taif University, Saudi Arabia; e Thule Institute, University of the Arctic, Oulu FI-90014, Finland

**Keywords:** Pesticides, Risk assessment, in-vitro metabolism, Human variability, Cytochrome P450s, LC-MS

## Abstract

Furathiocarb is a carbamate insecticide detected in ecosystems. Its main metabolite carbofuran has been alluded to affect birth outcomes and disturb hormone levels in humans. The metabolism of furathiocarb in humans has not been characterized. The metabolism studies were performed using hepatic microsomes from ten donors and fifteen human cDNA-expressed CYPs. The initial screening and identification of the metabolites were performed by LC-TOF. Quantiﬁcations and fragmentations were performed by LC/MS-MS. Furathiocarb was metabolized to eight phase I metabolites via two general pathways, carbofuran metabolic pathway and furathiocarb oxidation pathway. Six metabolites in the carbofuran metabolic pathway (carbofuran, 3-hydroxycarbofuran, 3-ketocarbofuran, 3-keto-7-phenolcarbofuran, 3-hydroxy-7-phenolcarbofuran, and 7-phenolcarbofuran) were identified with the help of authentic standards. The two unidentiﬁed metabolites in the furathiocarb oxidation pathway are probably hydroxylated and sulfoxidated derivatives of furathiocarb. The carbofuran metabolic pathway was more predominant than the furathiocarb oxidation pathway, ratios ranged from 24- to 115-fold in a 10-donor panel of hepatic microsomes. On the basis of recombinant CYP studies, the carbofuran pathway was dominated by CYP3A4 (95.9%); contributions by CYP1A2 (1.3%) and CYP2B6 (2.0%) were minor. The minor furathiocarb oxidation pathway was catalyzed by CYP2C19 and CYP2D6 (hydroxylated/sulfoxidated metabolite A) and by CYP3A5, CYP3A4 and CYP2A6 (metabolite B). High and significant correlation between carbofuran metabolic pathway and CYP3A4 marker activities (midazolam-1'-hydroxylation and omeprazole-sulfoxidation) were observed. Ketoconazole, a CYP3A4-inhibitor, inhibited the carbofuran pathway by 32–86% and hydroxylated/sulfoxidated metabolite-B formations by 41–62%. The data suggest that in humans, the carbofuran metabolic pathway is dominant, and CYP3A4 is the major enzyme involved. These results provide useful scientific information for furathiocarb risk assessment in humans.

## Highlights

• Eight Phase I metabolites were detected by LC-TOF-MS/MS.
• The carbofuran pathway was more rapid than the furathiocarb oxidation pathway
• The carbofuran pathway was dominated by CYP3A4 (96%).
• Ketoconazole inhibited the carbofuran pathway by 32–86%.
• The findings provide useful scientific information for furathiocarb risk assessment in humans.

## Graphical Abstract

**Figure:**
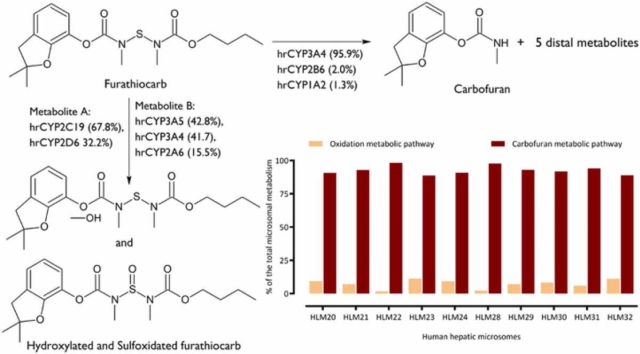

## 1 Introduction

Furathiocarb belongs to the carbamate class of insecticides, which also contains carbosulfan, benfuracarb, and benthiocarb. In terms of biotransformations, they are all converted to carbofuran, which is suggested to be the active insecticide [45]. While furathiocarb capacity to inhibit AChE directly cannot be excluded, carbofuran is a more AChE potent inhibitor. Both compounds bind to the acyl binding pocket via their carbamoyl moiety. Increasing the length of the carbamoyl moiety, as furathiocarb relative to carbofuran, decreases the AChE inhibition constant drastically [43]. Carbofuran, 3–hydroxycarbofuran, and 3–ketocarbofuran were detected as furathiocarb metabolites in animals [45]. Reports on furathiocarb biotransformation products in humans are limited to acute fatal poisoning cases, in which the more toxic carbofuran was not measured simultaneously [26]. However, urinary 3-hydroxycarbofuran is used as a main degradation metabolite of carbamates to assess exposure in epidemiological studies [13]; [14]; [18]. 3-Hydroxycarbofuran is rapidly eliminated into urine and reﬂects recent exposure [18], [63]. 3-hydroxycarbofuran was detected in urine samples collected during pregnancy from women living in the Salinas Valley of California [13] as well as in the National Health and Nutrition Examination Survey (NHANES) (1999–2002) [15]. It was detected in two urine samples and one fecal sample (n = 136, ages of 1.0 and 4.2 years) from healthy children in the UK [23]. 3-hydroxycarbofuran was also widely detected in urine from pregnant women from the Sheyang Birth Cohort (China). Urinary concentrations varied from 0.01 to 395.40 μg/L (n = 1100) and prenatal exposure was associated with adverse effects on fetal development [65].

Recently, several reports have described the use of liquid chromatography coupled to MS detectors (LC-MS/MS) in multiresidue analysis of pesticides, including carbamates, in raw coffee [44], melliferous weeds and agricultural crops [22], and apples [32] as well as in loggerhead turtles [36]. The gas chromatographic–tandem mass spectrometric method (GC-MS/MS) was employed for the determination of carbofuran and its distal metabolites in applicators’ urine [41] as well as the determination of 3-hydroxy carbofuran in maternal urine [65]. Unchanged furathiocarb was detected and quantified using TLC, GC/MS, and GC equipped with a nitrogen-phosphorus detector (GC-NPD) in human gastric contents [26]. Furathiocarb was detected also by LC in animal tissues [30], [31]. In our earlier studies, LC/TOF-MS and LC/MS–MS were used for metabolite identiﬁcation and quantiﬁcation of carbamate insecticides, carbosulfan and benfuracarb, biotransformation in mammalian in vitro hepatic models [2], [4].

Cytochrome P450 enzymes (CYPs) catalyze the biotransformation of a wide range of endogenous fatty acids, steroids, and lipophilic xenobiotics. CYPs are found in a variety of tissues, including kidney, lung, the gastrointestinal tract, skin, brain and nasal mucosa, and in high concentrations in the liver [39], [40], [64]. Variability in CYP activity within the human population is well established, and it is the one of the primary determinants of variability in xenobiotic metabolism [21], [29]. Human CYP3A4, the major enzyme involved in the in vitro bioactivation of both carbosulfan and benfuracarb to carbofuran, and CYP3A4-catalyzed biotransformation is the primary source of interindividual differences.

Furathiocarb biotransformation in humans has not been yet characterized in detail. The characterization and identification of metabolites detected in human hepatic in vitro system would enable toxicological evaluation of metabolites of concern which could be detected in different environmental matrices and human biomonitoring as well. Data emerging from kinetic studies may be of value for developing PBPK modelling and validating in vitro test models to predict and anticipate metabolic profiles in human in vivo [16]. Therefore, the study aimed to investigate the human variability, restricted to a panel of human hepatic microsomes from ten donors, of furathiocarb biotransformation in human hepatic microsomes. Furathiocarb biotransformation in human hepatic microsomes was systematically characterized by metabolic profiling, kinetics analysis, and inhibition assays. Additionally, human CYP isoforms involved in furathiocarb biotransformation were characterized by cDNA-expressed isoforms.

## 2 Materials and methods

Detailed information on various items in Materials and Methods is presented in Supplementary materials.

### 2.1 Chemicals

Furathiocarb and its metabolites, except the hydroxylated and sulfoxidated metabolites, were purchased from ChemService (West Chester, PA, USA). Solvents, HPLC-grade, were purchased from Labscan (Dublin, Ireland) and Rathburn (Walkerburn, UK). Other chemicals were purchased from Sigma Chemical Company (St. Louis, MO, USA)) and were of the highest purity available.

### 2.2 Human hepatic microsomes and cDNA-expressed CYPs

the Ethics Committee of the Medical Faculty of the University of Oulu, Finland approved the collection of human hepatic surplus from organ donors. Detailed characteristics of the collected samples are presented in Supplementary materials. Cytochrome P450 enzyme activity characterizations and incubations were performed within 12 months (Detailed information are available as Supplementary materials). All Baculovirus insect cell expressed human CYPs were obtained from BD Biosciences Discovery Labware (Bedford, MA).

### 2.3 Metabolite identiﬁcation

The incubation mixture (ﬁnal volume of 200 µl of 0.1 M phosphate buffer) contained 50 and 100 µM furathiocarb, 0.15 mg pooled liver microsomal protein (n = 10), and 1 mM NADPH. The reaction was initiated by NADPH after a 2 min pre-incubation at + 37 °C and was incubated for 30 min at + 37 °C. The reaction stopped with ice-cold acetonitrile containing an internal standard.

Furathiocarb was incubated with recombinant CYPs (rCYP). The incubation mixture contained rCYPs (50 pmol CYP per ml), 100 µM furathiocarb, and 1 mM NADPH in 200 µl 0.1 M phosphate buffer (pH 7.4). Incubation procedures and chromatographic separation are presented in Supplementary materials.

### 2.4 Mass spectrometry

**Fig. 1:**
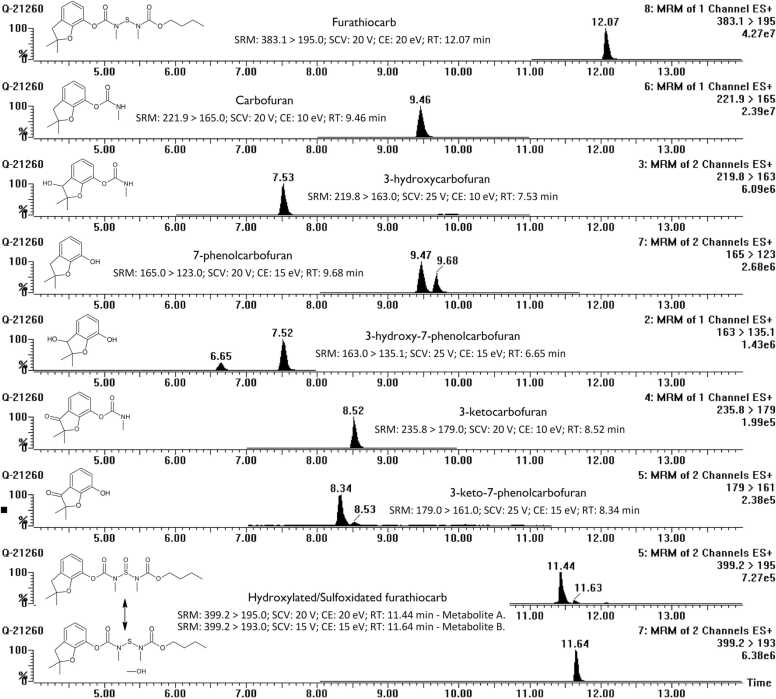
Extracted mass chromatograms of furathiocarb metabolites formed by in vitro incubation with mammalian hepatic microsomes. Furathiocarb (ﬁnal concentration 300 µM) was incubated with hepatic microsomes (0.15 mg protein) in the presence of NADPH in 0.1 M phosphate buffer at pH 7.4 for 60 min 3-hydroxycarbofuran, 3-hydroxy-7-phenolcarbofuran were quantified as the protonated dehydrated molecule [M−H2O+H]+ due to significant in-source fragmentation. Six metabolites (carbofuran, 3-hydroxycarbofuran, 3-ketocarbofuran, 3-keto-7-phenolcarbofuran, 3-hydroxy-7-phenolcarbofuran, and 7-phenolcarbofuran) were identified with the help of authentic standards. Analytical standards were not available for the presumed furathiocarb hydroxylated and sulfoxidated metabolites and they were tentatively identified on the basis of exact masses and fragmentation patterns and quantitated by using the calibration curve of furathiocarb, assuming their responses to be approximately equal. The lower limit of quantitation was 0.5 μM for all compounds. To ensure the quality of the analysis, external standards were measured in the beginning, middle, and end of the experiment. Carbaryl was used as an internal standard (SRM: 202.00 > 145.00; SCV: 25 V; CE: 15 eV; RT: 9.8 min). SRM; selected reaction monitoring; SCV: sample cone voltage (V); CE: collision energy (eV); RT: retention time. * Hydroxylation may take place on the carbamate N-methyl group, on an alkyl substituent, or on the aromatic ring itself.

Present and accurate mass measurements were carried out by Micromass LCT - TOF-MS (Micromass, Altrincham, UK) equipped with a Z-Spray ionization source. Micromass Quattro II triple quadrupole instrument equipped with a Z-spray ionization source was employed for the quantification and fragmentation measurements. Multiple reaction monitoring (MRM), collision energies, and sample cone voltages for metabolites and the internal standard are described in detail in Fig. 1.

### 2.5 Kinetic studies

Enzyme kinetic parameters of both the microsomal samples and rCYP enzymes were measured. The incubation mixture contained furathiocarb (2.5, 10, 25, 50, 150 and 300 µM). Incubation times were 20 and 30 min for microsomal samples and rCYPs, respectively. Otherwise incubation mixtures and methods were the same as in metabolite identification. The rate of sum of furathiocarb metabolite formation was linear at least up to 0.15 mg protein/ml and 30 min incubation time in the standard experimental conditions.

### 2.6 Correlation with model CYP substrate activities

A set of ten livers was used to assess the metabolism of furathiocarb in the individual livers as well as to correlate the activities with a model CYP substrate. (Detailed information is available as Supplementary materials).

### 2.7 Ketoconazole interactions

The inhibitory effects of ketoconazole, a known CYP3A4 isoform-selective inhibitor, on the formation of carbofuran were evaluated. Formation rates of metabolites were determined in the presence or absence of ketoconazole 100 µM.

### 2.8 Human CYP-furathiocarb inhibitory interactions by N-in-one assay incubation

N-in-one (“cocktail”) assay for measuring major CYP activities simultaneously [1], [55], [56] was employed to investigate human CYP-furathiocarb inhibitory interactions in pooled human hepatic microsomes. Furathiocarb (ﬁnal concentrations in the incubation mixture were 1, 5, 25, 50, and 100 µM) was added to the incubation mixture. The N-in-one was used earlier for the study of pesticide interactions and the details of the incubations with furathiocarb are described in Supplementary materials.

### 2.9 Statistical analysis

The kinetic parameters *V*_*max*_ and *K*_*m*_ were calculated by nonlinear regression. Bivariate linear Pearson’s correlation coefﬁcients (r2) were calculated between metabolite formations and model activities in the livers. GraphPad Prism 9 Software (Inc., San Diego, CA) Prism 9.0.0 (San Diego, CA) was used for kinetic parameters and correlation coefﬁcients analysis. The IC_50_ values were determined by linear regression analysis using OriginPro 2020 (MicroCal Software, Inc., Northampton, MA.

## 3 Results

### 3.1 Profiles of furathiocarb metabolites in human hepatic microsomes

Metabolic profile of furathiocarb in human pooled hepatic microsomes (0.15 mg protein) was studied in the presence of NADPH in 0.1 M phosphate buffer at pH 7.4 for 60 min. Incubates were analyzed by LC-TOF-MS. After optimization, the optimal collision energy for most detected compounds was at 10–15 eV for product ion analysis. For furathiocarb and its hydroxy/sulfoxide-furathiocarb metabolite, a higher collision energy (20 eV) was found to be optimal for ion analysis. Applying a higher collision energy did not significantly increase the appearance of ions in the spectrum. Due to signiﬁcant in-source fragmentation, two metabolites, 3-hydroxycarbofuran and 3-hydroxy-7-phenolcarbofuran, were quantiﬁed as the protonated dehydrated molecules [M−H_2_O+H]^+^. Six metabolites (carbofuran, 3-hydroxycarbofuran, 3-ketocarbofuran, 3-keto-7-phenolcarbofuran, 3-hydroxy-7-phenolcarbofuran, and 7-phenolcarbofuran) were identified with the help of standards out of eight metabolites detected from the extracted mass chromatograms (Fig. 1). Two unidentiﬁed metabolite peaks in the chromatogram are probably either hydroxylated or sulfoxidated derivatives of furathiocarb. Incubations with human hepatic homogenate fortified also with UDP-glucuronic acid, a cofactor for the phase II enzyme UDP-glucuronosyl transferase (UGT), were performed, but no phase II metabolites were detected (data not illustrated). However, further elucidation of possible phase II metabolic routes would require further studies with the help of hepatocytes and/or modification of incubation conditions more suitable for the detection of conjugates.

Carbofuran metabolic pathway involved the parent carbofuran with five more distal metabolites; these were added to provide a sum of carbofuran metabolic pathway. More distal metabolic steps away from carbofuran were not further characterized, so the enzymes catalysing the formation of each one of these distal conversions are not known. However, the addition of 5 metabolites formed provides a more comprehensive estimate of the carbofuran pathway. Two tentatively identified hydroxylated/sulfoxidated metabolites constituted the furathiocarb oxidative pathway.

### 3.2 Furathiocarb biotransformation in individual human hepatic microsomes

The kinetics of furathiocarb biotransformation in individual human hepatic microsomes (n = 10 donors) were characterized using a wide range of furathiocarb concentrations (2.5 – 300 µM) for the formation of carbofuran metabolic pathway (Table 1). Individual 30 (HLM30) had the highest afﬁnity, corresponding to the lowest *K*_*m*_ (25.9 µM). Individual 22 (HLM 22) had the lowest afﬁnity, corresponding to the highest *K*_*m*_ (71.5 µM), and the highest capacity corresponding to the highest *V*_*max*_ (44.5 nmol/(mg protein * min)). The lowest capacity (*V*_*max*_ = 15.5 nmol/(mg protein*min)) was measured from individuals HLM31 and HLM32. The highest *CL*_*int*_ rate was observed with HML28, while individual HML31 had the lowest (244 µl/(mg protein * min)).

**Table 1:** Carbofuran metabolic pathway and furathiocarb oxidation pathway kinetic parameters obtained with ten human liver microsomes^a^.

*Human liver microsomes*	*Sum of carbofuran metabolic pathway (Predominant)*			*Sum of furathiocarb oxidation pathways (minor pathway)*			*carbofuran pathway / oxidation pathways*
	*V*_*max*_	*K*_*m*_	*CL*_*int*_	*V*_*max*_	*K*_*m*_	*CL*_*int*_	
	nmol/ (mg protein min)	µM	µl/ (mg protein min)	nmol/ (mg protein min)	µM	µl/ (mg protein min)	
HLM20	19.6 ± 1.7	63.2 ± 16.3	310.4	4.3 ± 0.8	343.1 ± 127.6	12.63	24.5
HLM21	22.6 ± 2.5	66.7 ± 20.6	339.0	4.1 ± 1.1	453.1 ± 215.2	9.22	36.8
HLM22	44.5 ± 5.1	71.8 ± 21.7	619.7	nd	nd	nd	–
HLM23	17.4 ± 1.6	50.0 ± 14.1	348.6	3.5 ± 7.3	260.4 ± 116.9	13.48	25.9
HLM24	21.9 ± 2.1	54.3 ± 16.1	404.5	5.1 ± 1.3	409.5 ± 196.6	12.25	33.0
HLM28	43.6 ± 4.7	55.8 ± 18.0	779.6	11.5 ± 6.4	1706 ± 852	6.75	115.4
HLM29	24.3 ± 2.5	64.2 ± 18.9	378.2	5.3 ± 1.4	495.4 ± 237.3	10.73	35.2
HLM30	18.2 ± 1.8	25.9 ± 9.5	701.2	5.1 ± 1.3	455.5 ± 208.2	10.99	63.8
HLM31	15.5 ± 1.1	63.3 ± 13.4	244.3	1.7 ± 0.3	238.8 ± 79.5	7.00	34.9
HLM32	15.5 ± 1.4	38.8 ± 11.3	399.9	3.7 ± 0.6	280.4 ± 101.9	13.04	30.6

a Each value represents the mean ± std. error of three determinations.

Additionally, kinetic parameters were studied for furathiocarb oxidation pathways (Table 1). Kinetics for the sum of the two metabolites showed HLM31 displayed the highest afﬁnity (*K*_*m*_ values 238.8 µM) and lowest capacity (*V*_*max*_ values 1.7 nmol/(mg protein * min)). HLM28 displayed the highest afﬁnity capacity (*V*_*max*_ = 11.5 nmol/(mg protein * min)). The catalytic efﬁciencies *CL*_*int*_ for HLM28 and HLM31 (6.75 and 7.0 µl/(mg protein * min)) were the lowest, while HLM32 had the highest efﬁciency (13.04 µl/(mg protein * min)) (Table 1). Results showed limited variabilities, restricted to hepatic microsomes from ten donors, in carbofuran metabolic pathway kinetic parameters (2.7, 2.8, and 3.1-fold) in *k*_*m*_, *V*_*max*_ and *Cl*_*int*_ values, respectively.

### 3.3 Characterization of distal metabolite formation

**Fig. 2:**
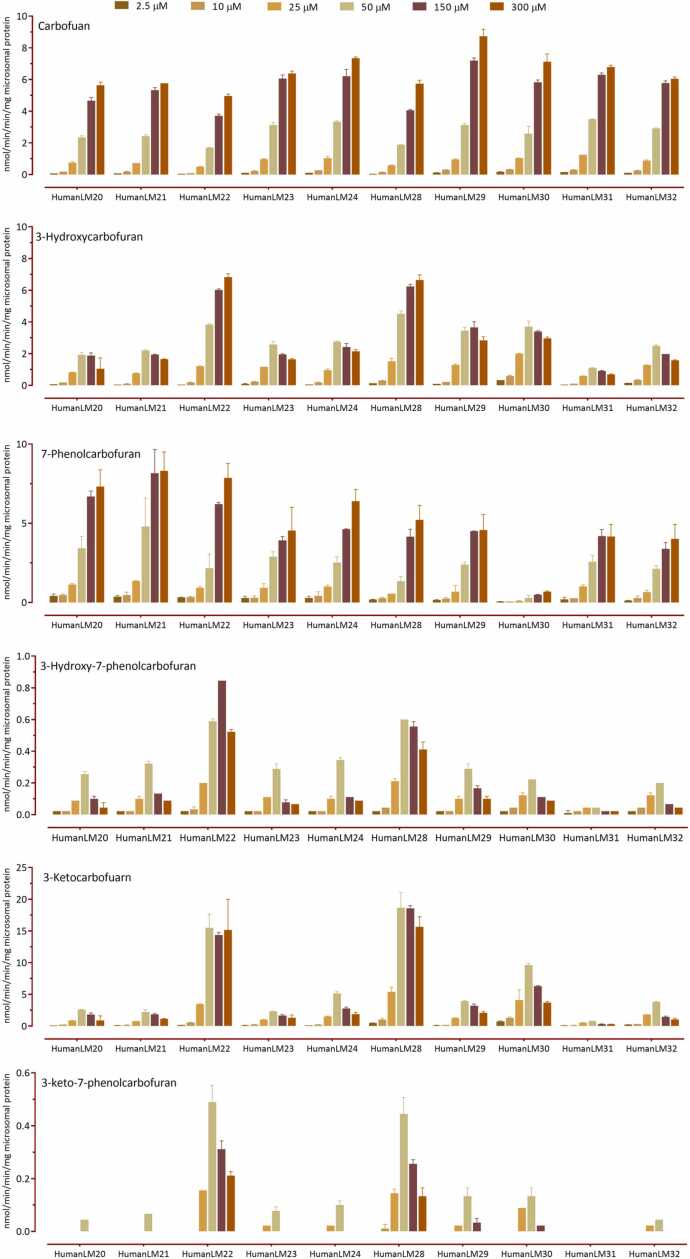
Interindividual variability in distal furathiocarb metabolite formation rates in human hepatic microsomes from 10 individual donors. Each column represents the mean ± S.D of three separate determinations.

The variations in distal metabolite formation rates was further characterized (Fig. 2). HLM29 had the highest carbofuran formation rates, while HLM20 and HLM22 exhibited the lowest formation rates. HLM22 and HLM28 had the highest 3-hydroxycarbofuran, 3-hydroxy-7-phenolcarbofuran, 3- ketocarbofuran, and 3-keto-7-phenolcarbofuran formation rates. The 3-hydroxycarbofuran formation rate varied from 0.68 to 6.83 nmol/(mg protein min) at 300 µM furathiocarb, displaying 10-fold variation. The variations between the highest and lowest formation rate were 2-fold for 7-phenolcarbofuran (4.5–8.3 nmol/(mg protein min)), 23-fold for 3-hydroxy-7-phenolcarbofuran (0.02–0.52 nmol/(mg protein min)), and 54.4-fold for 3-ketocarbofuran (0.28–15.6 nmol/(mg protein min)) at 300 µM furathiocarb. 3-keto-7-phenolcarbofuran amounts were below the limits of quantiﬁcation in HLM31 with all furathiocarb concentrations used. 3-keto-7-phenolcarbofuran is a distal metabolite of both 3-ketocarbofuran and 3-hydroxy-7-phenolcarbofuran, and individual HLM31 had the lowest amounts of those metabolite formation rates.

### 3.4 Furathiocarb biotransformation by human CYP isoforms

#### 3.4.1 Kinetics of furathiocarb metabolism in human cDNA-expressed cytochrome P450s

**Fig. 3:**
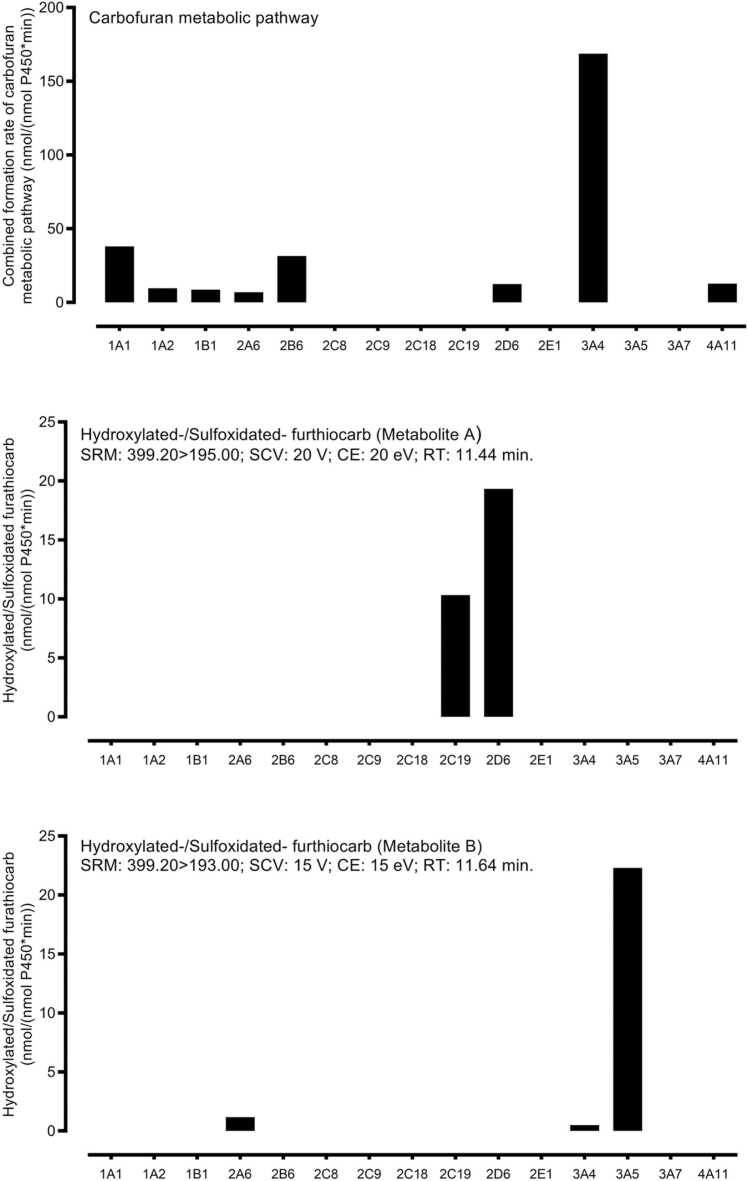
Screening assay with human cDNA-expressed cytochrome P450s. 10 pmol/ml cDNA-expressed cytochrome P450s was incubated with 100 µM furathiocarb benfuracarb at 37 °C for 20 min. The incubation was performed in the presence of NADPH. Recombinant P450 isozymes used in the present study were co-expressed with cytochrome P450 reductase and/or cytochrome B5. The amounts of cytochrome P450 reductase and cytochrome B5 of the recombinant P450 isozymes used in the present study were 140–2100 nmol/min/mg protein and 190–1100 pmol/mg protein, respectively. Results are expressed as the metabolite formation rates of duplicate samples.

To determine the human CYP isoforms metabolizing furathiocarb, several approaches were followed. These included cDNA-expressed human CYPs, correlation with marker activities in human hepatic microsomes, and the specific CYP isoform inhibitor approach. Upon incubation with 15 human cDNA-expressed P450 isoforms and NADPH, carbofuran metabolic pathway was found to be mediated by CYP1A1/2, 1B1, 2A6, 2B6, 2D6, 3A4 and 4A11. CYP3A4 appeared to be the most active CYP in carbofuran metabolic pathway formation. Preliminary results from the screen assay also showed that a different CYP was involved in furathiocarb sulfoxidation to either hydroxylated or sulfoxidated derivatives of furathiocarb (Fig. 3).

Detailed kinetic analysis, based on the preliminary screening assays, showed that CYP2B6 and CYP3A4 had the highest afﬁnity besides CYP1A2 and CYP2D6, corresponding to the lowest *K*_*m*_, whereas CYP2A6 had the lowest in the case of the activation pathway. CYP3A4 had the highest capacity, while CYP1A2 and CYP2D6 had the lowest capacity. The *CL*_*int*_ values showed that CYP3A4 was the most efﬁcient isoform for carbofuran metabolic pathway, whereas CYP2A6 was the least efﬁcient.

In the case of furathiocarb oxidation pathway, different rCYPs were responsible for furathiocarb biotransformation to hydroxylated or sulfoxidated metabolites. *Cl*_*int*_ values showed that CYP2C19 was more efficient than CYP2D6 in the formation of the hydroxylated/sulfoxidated metabolite detected at RT 11.4 min (metabolite A). CYP3A5 was more efficient than CYP2A6 and CYP3A4 in the formation of the hydroxylated/sulfoxidated metabolite detected at 11.7 min (metabolite B) (Table 2).

**Table 2:** Kinetic parameters of furathiocarb metabolic pathways baculovirus-infected insect cells expressing human cytochrome P450 enzymes^a^.

P450 isoforms	*V*_*max*_	*K*_*m*_	*CL*_*int*_	Relative contribution^b^
	nmol/ (nmol P450 min)	µM	µl/ (nmol P450 min)	
**Carbofuran metabolic pathway**^c^				
CYP1A1	89.2 ± 13.5	49.8 ± 24.6	1790	n.d
CYP1A2	11.8 ± 1.3	16.7 ± 7.5	711	1.3
CYP1B1	26.5 ± 3.1	69.7 ± 24.1	380	0.0
CYP2A6	60.7 ± 20.0	284.3 ± 196	214	0.3
CYP2B6	26.4 ± 4.2	9.7 ± 3.9	2704	2.0
CYP2D6	15.5 ± 2.1	18.7 ± 5.8	808	0.5
CYP3A4	151.3 ± 12.3	6.9 ± 2.5	21915	95.9
CYP4A11	30.1 ± 1.6	58.4 ± 6.9	516	n.d

**Hydroxylated/Sulfoxidated furathiocarb (Metabolite A)** **SRM: 399.20 > 195.00; SCV: 20 V; CE: 40 eV; RT: 11.44 min**				
CYP2C19	10.1 ± 0.9	5.6 ± 2.4	1783	67.8
CYP2D6	28.6 ± 2.1	38.6 ± 9.6	741	32.2

**Hydroxylated/Sulfoxidated furathiocarb (Metabolite B)** **SRM: 399.20 > 193.00; SCV: 15 V; CE: 15 eV; RT: 11.64 min**				
CYP2A6	53.1 ± 14.1	274.8 ± 55.2	193	15.5
CYP3A4	69.7 ± 16.2	463.6 ± 188.0	150	41.7
CYP3A5	31.3 ± 1.7	37.1 ± 8.7	844	42.8

n.d not determined.

a *V*_*max*_ and *K*_*m*_ values represent means ± S.E. of three determinations.

b Mean human hepatic microsomal protein amounts of P450 enzymes are taken from Achour et al. [5].

c Combined formation rates of all the metabolites of the carbofuran pathway were used for the calculation of kinetic parameters.

#### 3.4.2 Correlation analysis of marker activities in human hepatic microsomes

Individual CYP marker activities from ten human hepatic microsomes and incubations were performed at the same time and correlation studies are presented in Table 3. High and significant correlations between the carbofuran metabolic pathway and CYP3A4 (1-OH-MDZ and SO2-OME) marker activities as well as CYP2C19 (5-OH-OME) were observed. No other significant correlations were seen with other CYP marker activities.

**Table 3:** Linear correlation coefficients for furathiocarb metabolites with CYP-mediated activities in a panel of human hepatic microsomes (*n* = 10).

Enzyme	Reaction	Carbofuran metabolic pathway	-OH/SO4 furathiocarb Metabolite A	-OH/SO4 furathiocarb Metabolite B
CYP1A1/2	7-Ethoxyresorufin-O-deethylation (EROD)	0.2493		
CYP2A6	Coumarin-7-hydroxylation (OH-COU)	0.2303		0.1259
CYP2B6	Bupropion hydroxylation (OH-BUP)	0.0023		
CYP2C8	Amodiaquine-desethylation (deEt-AMO)	0.1814		
CYP2C9	Tolbutamide hydroxylation (OH-TOL)	0.1126		
CYP2C19	Omeprazole-5- hydroxylation (5-OH-OME)	0.9074 **	0.4683	
CYP2D6	Dextromethorphan-*O*- desmethylation (*O*-dem-DEX)	0.0059		
CYP2E1	Chlorzoxazone-6-hydroxylation (OH-CLZ)	0.1101		
CYP3A4	Midazolam-1'-hydroxylation (1-OH-MDZ)	0.8513 *		0.3282
CYP3A4	Omeprazole sulfoxidation (SO2-OME)	0.9632 **		0.2793

1 Quantification of furathiocarb biotransformation in vitro by ten individual human liver microsomes was performed with 50 μM furathiocarb for carbofuran metabolic pathway and 100 μM furathiocarb for furathiocarb metabolite A and B and a 20-min incubation time. Metabolites A and B were detected in 4 individual human liver microsomes at 50 μM furathiocarb and did not permit correlation studies.

* , P value = 0.0001

** , P value < 0.0001

#### 3.4.3 Inhibition of furathiocarb biotransformation by ketoconazole

**Fig. 4:**
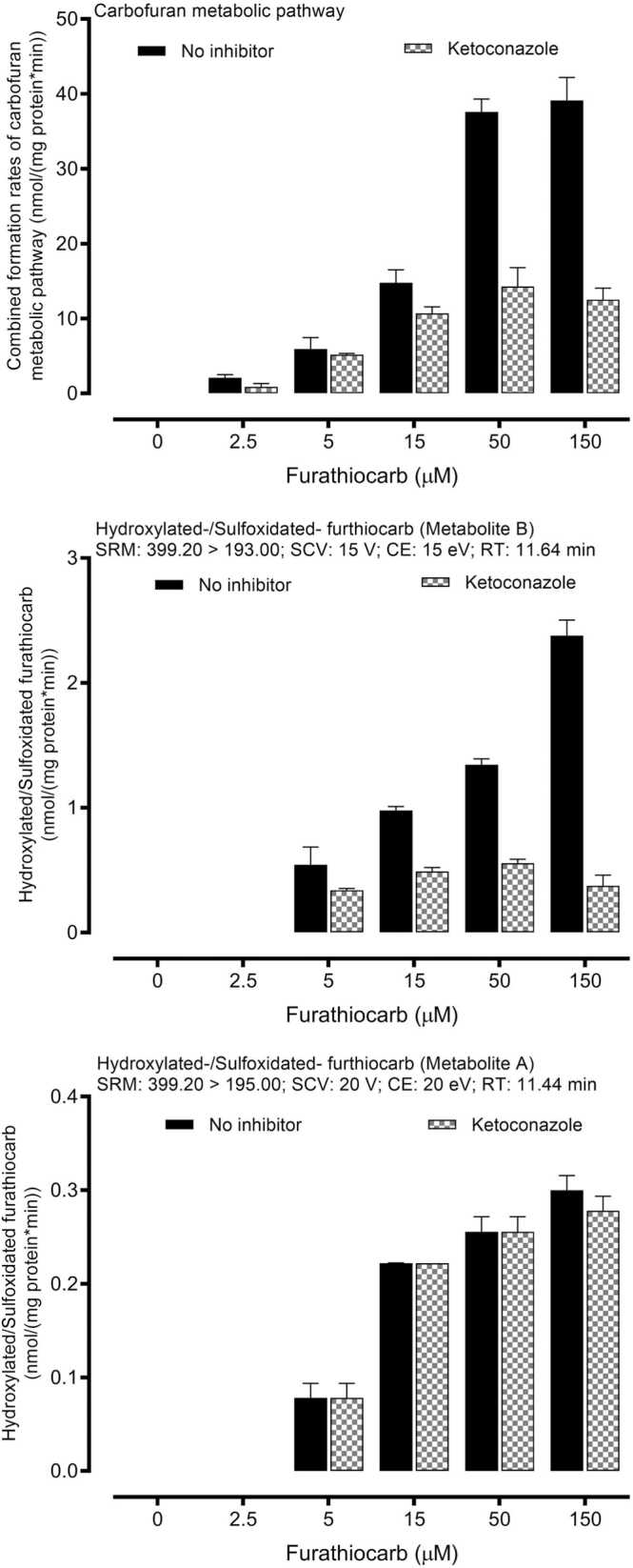
**:** Effect of ketoconazole on furathiocarb metabolites formation rates. Pooled human hepatic microsomes (0.15 mg protein/ml) from 10 individual donors were incubated with various furathiocarb concentrations at 37 °C for 20 min in the presence of the CYP3A4 isoform-selective inhibitor, ketoconazole. Each point represents the mean ± S.D of three separate determinations.

Ketoconazole, a CYP3A4 isoform-specific inhibitor (Fig. 4), was incubated with different furathiocarb concentrations in pooled human hepatic microsomes. Furathiocarb was a substrate for several CYPs, and ketoconazole could significantly inhibit the overall metabolism of furathiocarb. Ketoconazole inhibited the carbofuran metabolic pathway formation by 32–86% and to a lesser degree hydroxylated/sulfoxidated metabolite formation (metabolite B) (41–62%). Ketoconazole showed no effects on the formation of metabolite A. The results collectively confirmed the role of CYP3A4 in furathiocarb biotransformation in humans.

#### 3.4.4 Potential interactions with the human hepatic P450 approach

CYP inhibitory potential was studied by N-in-one assay in pooled human hepatic microsomes using a furathiocarb concentration range of 1–100 µM to evaluate CYP-mediated metabolic interactions. The IC_50_ values were in the range of 16.5 – 18.5 µM for CYP1A2 (OH-MEL), CYP2B6 (OH-BUP), CYP2C9 (OH-TOL), and CYP2C19 (5-OH-OME). Other IC_50_ values were above 100 µM, indicating very low affinities.

## 4 Discussion

Metabolic profiles of furathiocarb in pooled human hepatic microsomes showed that furathiocarb was metabolized to eight phase I metabolites. Six metabolites, representing the carbofuran (metabolic) pathway (carbofuran, 3-hydroxycarbofuran, 3-ketocarbofuran, 3-keto-7-phenolcarbofuran, 3-hydroxy-7-phenolcarbofuran, and 7-phenolcarbofuran) were identified with the help of authentic standards. Two unidentiﬁed metabolites, representing the detoxification pathway, are probably either hydroxylated or sulfoxidated derivatives of furathiocarb. Hydroxylation may take place on the carbamate N-methyl group, on an alkyl substituent, or on the aromatic ring itself. The hydroxylation at the phenyl ring and at the N-methyl moiety were reported in the metabolism of the structurally related compound bendiocarb [45]. Moreover, predominant metabolic routes for carbofuran in mammals are via ring and N-methyl hydroxylation followed by conjugation reactions [45]. Ring hydroxylation was reported for carbofuran in vitro metabolism by human, mouse, and rat cytochrome P450 [58]. Hydroxylation at the N-methyl group of carbofuran was detected in rat liver, insects, and plants [45]. Three N-methyl hydroxy metabolites, but in minor amounts, were detected in insects and plants [57]. Additionally, ring hydroxylation was reported with benfuracarb metabolism in plants. Metabolites found in plants, mammals, and insects are often similar and differ only in the nature of conjugates [45], [60]. On the other hand, sulfoxidation was reported with the structurally related compounds carbosulfan and benfuracarb. Carbosulfan sulfoxidation was detected with an unknown method in human, mouse, and rat liver microsomes [24]. Both carbosulfan and benfuracarb sulfoxidation were detected with LC-MS/MS in several mammalian hepatic microsomes [2], [4]. Hydroxylated and sulfoxidated derivatives of furathiocarb (Metabolite A and Metabolite B) were tentatively identified on the basis of exact masses and fragmentation patterns and they were quantitated by using the calibration curve of furathiocarb, assuming their responses to be approximately equal.

It is worth mentioning that in our study, no non-enzymatic hydrolysis of the reference standards was detectable. Likewise, a high stability of reference standard solutions was reported earlier [66]. Therefore, it is concluded that distal metabolites detected in our study appeared by enzymatic biotransformation, very likely mediated by cytochrome P450 enzymes, because the formation was NADPH-dependent, and reactions are typical and frequent for P450-catalysis. Furthermore, illustrated interindividual variability as well as interspecies variations (Abass et al. a companion article submitted) in the formation of furathiocarb distal metabolites support this ﬁnding as well. On the basis of our findings and earlier publications on related carbamates [2], [4], the main metabolic pathways of furathiocarb in mammalian hepatic microsomes in vitro were the hydroxylation and sulfoxidation of furathiocarb (metabolite A and B) and the cleavage of the nitrogen–sulfur bond to give the carbofuran metabolic pathway.

Biotransformation kinetics showed that the carbofuran metabolic pathway is dominant. The ratios between carbofuran metabolic pathway/oxidation in *CL*_*int*_ were the highest in HLM28 (115-fold) followed by HLM30 (64-fold), while HLM20 had the lowest differences (24.5-fold). The up to 115-fold variation, restricted to a panel of human hepatic microsomes from ten donors, may be a signiﬁcant determinant of furathiocarb potent toxicity in humans, because the carbofuran metabolic pathway, contains metabolites more potent than the parent compound. Furathiocarb neurotoxicity depends on its bioactivation to carbofuran [45]. Carbofuran is assigned to a toxic class of 1b according to WHO classification of pesticides by hazard [6]; [54]. Several carbofuran-related homicides and suicides have been reported [54]. Carbofuran can cross the placental barrier and cause several developmental disorders in adult rats and dogs [34]; Mishra et al. 1995; Mishra et al. 1997; [61]. Studies on rodents showed that exposure to carbofuran may cause physical and neurological disorders and impact endocrine homeostasis and hormone balance during the prenatal and postanal periods [7], [19], [50]. In humans, parental exposure to carbofuran may be associated with adverse birth outcomes [8], [51], [62]. Carbofuran concentration in cord blood was negatively associated with head circumference of infants from a New Jersey cohort of pregnant women [8].

Reports on acute/chronic exposure to furathiocarb are limited to one report [26]. Furathiocarb was detected in gastric contents and quantified in the blood of 7 cases of acute fatalities, but its main and distal metabolites were not studied. The authors reported wide variabilities in furathiocarb concentrations in the blood (0.1–21.6 µg/ml (n = 7, average 3.6 µg/ml)) due to differences in ingested doses and time intervals between ingestion and death [26].

Available publications on carbofuran accidental [47] and suicidal poisoning [6]; [17]; [42] did not report main or distal metabolite concentrations. However, carbofuran and 3-hydroxycarbofuran were detected in blood, bile, liver, and stomach in seven carbofuran fatal cases by GC-MS [46]. Only 3-hydroxycarbofuran was identified in one case in blood. 3-hydroxycarbofuran is used as a biomarker in epidemiological studies to assess exposure to certain carbamates, i.e. furathiocarb [13]; [14]; [18].

Screening assays and kinetics obtained with cDNA-expressed human CYPs isoforms showed that the two proposed hydroxylated/sulfoxidated metabolites are not the same since they were generated by different isoforms. CYP2C19 and CYP2D6 mediated the formation of the metabolite detected at RT 11.4 min (hydroxylated/sulfoxidated metabolite, named metabolite A). CYP2C19 mediated sulfoxidation of structurally diverse pesticides such as endosulfan-α [12], Fenthion [28], Fipronil [53], methiocarb, sulprofos and phorate [59]. CYP2C19 also mediated the oxidative metabolism of mainly pyrethroids pesticides i.e. bifenthrin, bioresmethrin, cis-permethrin, cypermethrin, s-bioallethrin; resmethrin, β-cyfluthrin, and λ-cyhalothrin [49], and esfenvalerate and deltamethrin [20]. Additionally, CYP2C19 mediated aromatic hydroxylation of the carbamate insecticides carbaryl and carbofuran [52], [58]. CYP2D6 has a role in mediating the sulfoxidation reaction of disulfoton, methiocarb, and sulprofos [58] as well as carbaryl aromatic hydroxylation [52].

Three human rCYPs mediated the formation of the hydroxylated/sulfoxidated metabolite detected at RT 11.7 min (named metabolite B). CYP3A5 had the highest *Cl*_*int*_ values, 4.3-fold and 5.6-fold higher than CYP2A6 and CYP3A4, respectively. It has been reported that CYP3A5 mediated the sulfoxidation of endosulfan-α and endosulfan-β (S. [27] and sulprofos [58], as well as the aromatic hydroxylation of carbaryl [52] and the oxidative metabolism of deltamethrin and esfenvalerate [20]. CYP2A6 was involved in the aromatic hydroxylation and methyl oxidation of carbaryl [52] and imidazolidine oxidation of imidacloprid [48]. Likewise, earlier findings showed CYP3A4 could mediate both sulfoxidation and hydroxylation reactions in structurally diverse pesticides. CYP3A4 was involved in the sulfoxidation of ametryne [25], disulfoton, disulfoton, methiocarb phorate, sulprofos [58], endosulfan-α and endosulfan-β (S. [27], fenthion [28] and fipronil [53]. CYP3A4 also mediated the hydroxylation reaction of diniconazole, epoxiconazole, fenbuconazole, hexaconazole, ipconazole, metconazole, paclobutrazole, triticonazole, and uniconazole [33], and the oxidative metabolism of bioresmethrin, cis-permethrin, cypermethrin, s-bioallethrin; resmethrin, β-cyfluthrin, and λ-cyhalothrin [49]. Although it is challenging to identify which metabolite is the hydroxylated or the sulfoxidated based on CYP-mediated reactions, it is obvious that the two unidentified metabolites are not the same.

Although in vitro kinetic studies provide vital information on the intrinsic clearance value of each CYP, it is important to consider the relative abundance of each CYP isoform in humans in vivo and estimate the relative contribution of individual CYP isoforms. For the carbofuran metabolic pathway, the relative contribution of different isoforms calculated based on *CL*_*int*_ and the mean average human hepatic microsomal protein [5] was dominated by CYP3A4 (95.9%) followed by CYP2B6 (2.0%) and CYP1A2 (1.3%). For the oxidation pathway, the relative contribution calculations showed the formation of the hydroxylated/sulfoxidated metabolite A was dominated by CYP2C19 (67.8%) followed by CYP2D6 (32.2%). CYP3A5, CYP3A4, and CYP2A6 dominated the formation of the hydroxylated/sulfoxidated metabolite B (42.8%, 41.7%, and 15.5%, respectively). CYP3A5 was 5.7-fold more efﬁcient (*Cl*_*int*_ 844 µl/(nmol P450 min)) than CYP3A4 (*Cl*_*int*_ 150 µl/(nmol P450 min)) for furathiocarb oxidation (metabolite B), their relative contributions to human in vivo clearance are roughly the same. CYP3A4 is the major isoform of CYPs expressed in human liver and gastrointestinal tract [5], [21], [39].

Levels of 3-hydroxycarbofuran in humans may reflect exposure to carbamate insecticides, including furathiocarb [63]. Our in vitro studies showed that in addition to 3-hydroxy carbofuran, 7-phenol carbofuran could be used as biomarkers to assess exposure to carbofuran-related carbamates insecticides. Data from individuals 22 and 28 indicated that 3-ketocarbofuran could be used as a biomarker as well. However, individuals 22 and 28 had the highest level of CYP3A4. In general, CYP catalytic activities in humans are widely varied and sometimes lead to different responses and metabolic capabilities of humans to toxicants and drugs. Large interindividual variabilities in CYP expression and activities in humans are apparent and may lead to different responses and susceptibilities to toxins and xenobiotics. Human exposure to environmental factors, contaminants, drug use, and nutritional status affect their metabolic capabilities and cytochrome P450 activities as well. Moreover, other factors, such as aging, genetic differences, and gender complicate the understanding and estimation of interindividual responses to xenobiotics [21], [40].

Furathiocarb potential interactions with the human CYP activities were studied by N-in-one screening assay. The N-in-one assay provides quick and comprehensive and evaluation of potential inhibitory interactions with CYP activities, but the assay does not provide data on the mechanism of inhibition. Carbosulfan and benfuracarb, carbamates insecticides with a similar structure as furathiocarb, were found to inhibit CYP3A4, with IC_50_ values of 11.2 µM and 14.8 µM (MDZ-OH) and 24.2 µM and 23.8 µM, respectively. Additionally, carbosulfan inhibited CYP2C9, 2C19 and CYP2D6 with IC_50_ values of 16 – 25 µM. On the other hand, organophosphate insecticides, rather than other pesticide groups, are known for their potent inhibitory interactions with CYPs. Chlorpyrifos potently inhibited several CYPs (1A1/2, 2B6, 2D6, and 3A4) with IC_50_ values of 2.9, 2.5, 3.3, and 4.0 µM, respectively [3]. The potent organophosphate-CYP interaction is due to the irreversible binding of the reactive sulphur released during CYP-mediated desulfuration to the heme iron of CYP [11], [35]. Our results showed that furathiocarb is not a potent CYP inhibitor. Indeed, internal doses from environmental exposure are crucial to cause CYP-furathiocarb interactions in vivo. It has been suggested, based on chlorpyrifos human plasma levels of chlorpyrifos as well as pharmacokinetic models in adults, children and agricultural workers, that concentrations of 10–50 µM in vitro are the most appropriate concentrations to mimic in vivo conditions of exposure to organophosphate pesticides mimic [10], [9]. Even though this was estimated for a particular group of pesticides, it is unlikely to achieve a high internal dose from environmental exposure to furathiocarb, and therefore, in vivo metabolic interactions with other xenobiotics or endobiotics are unlikely.

## 5 Conclusion

In summary, the data presented indicate that the hepatic biotransformation of furathiocarb in human liver microsomes occurs predominantly via nitrogen sulfur bond cleavage, leading to the carbofuran metabolic pathway, involving carbofuran and its distal metabolites. Reliable characterization of the metabolite profile is of considerable importance if metabolites unique to humans are identified in comparison with other species used in toxicological studies. Unique metabolites need specific attention when considering the relevance of in vivo animal studies to cover potential toxicity in humans. Furathiocarb metabolic profile in in-vitro human hepatic microsomes is in line with human in vivo findings. The use of carbofuran and 3-hydroxycarbofuran to assess exposure to furathiocarb and other related carbamates in epidemiological studies is justifiable. Although multiple hepatic CYP enzymes were involved in the biotransformation, the predominant CYP isoform involved in the formation of the carbofuran metabolic pathway was CYP3A4. The furathiocarb sulfoxidation pathway was relatively insignificant in furathiocarb metabolism in human liver microsomes. CYP2C19 followed by CYP2D9 catalyzed the formation of hydroxylated/sulfoxidated metabolite A. CYP3A5 followed by CYP3A4 and CYP2A6 were involved in the formation of hydroxylated/sulfoxidated metabolite B. Our findings are significant in revealing (at least tentatively) interindividual variability in humans and in contributing in a more general manner to toxicological evaluation and risk assessment.

## Funding

This research was supported by the European Union's Horizon 2020 program EDCMET (grant number 825762).

## CRediT authorship contribution statement

**Khaled Abass:** Conceptualization; Formal analysis, Visualization, Writing – original draft, Writing – review & editing. **Petri Reponen:** Writing – review & editing, Chemical analysis. **Olavi Pelkonen**: Conceptualization, Writing – original draft, Writing – review & editing.

## Declaration of Competing Interest

The authors declare that they have no known competing financial interests or personal relationships that could have appeared to influence the work reported in this paper.
