# Supplementary material for: Characterization of furathiocarb metabolism in in-vitro human liver microsomes and recombinant cytochrome P450 enzymes
Source: Toxicol Rep. 2022 Apr 1;9:679–89. doi: 10.1016/j.toxrep.2022.03.046 (PMC8989696; doi:10.1016/j.toxrep.2022.03.046)
Supplement: Supplementary file 1 — Supplementary material [file mmc1.docx]

**Characterization of furathiocarb metabolism in *in vitro* human liver microsomes and recombinant cytochrome P450 enzymes**

Khaled Abass^1,2,3^*, Petri Reponen^2^, Walaa F. Alsanie^4^, Arja Rautio^1,5^ and Olavi Pelkonen^2^

^1^ Arctic Health, Faculty of Medicine, P.O. Box 7300, FI-90014, University of Oulu, Finland.

^2^ Pharmacology and Toxicology Unit, Research Unit of Biomedicine, P.O. Box 5000, FI-90014 University of Oulu, Oulu, Finland.

^3^ Department of Pesticides, Menoufia University, P.O. Box 32511, Egypt.

^4^ Department of Clinical Laboratory Sciences, The Faculty of Applied Medical Sciences & Centre of Biomedical Sciences Research (CBSR), Taif University, Saudi Arabia.

^5^ Thule Institute, University of the Arctic, FI-90014 Oulu, Finland.

- 1. **Human liver homogenates and mammalian liver microsomes**

Human liver samples used in this study were obtained from the University Hospital of Oulu as surplus from organ donors. The collection of surplus tissue was approved by the Ethics Committee of the Medical Faculty of the University of Oulu, Finland. Human liver samples were of Caucasian race including 4 female and 6 male subjects between the ages of 21 and 62. Characteristics of the liver samples are presented in Table 1. The livers were transferred to ice immediately after the surgical excision and cut into pieces, snap-frozen in liquid nitrogen and stored at -80 °C. Human liver homogenate was prepared from livers of 10 individuals by homogenizing liver tissue in four volumes of 0.1 M phosphate buffer (pH 7.4), i.e. the homogenate contained 200 mg of liver tissues/ml. All microsomes were prepared by standard differential ultracentrifugation. Microsomes were prepared by standard differential ultracentrifugation (Pelkonen et al., 1974). The final microsomal pellet was suspended in 100 mM phosphate buffer, pH 7.4. Protein content was determined by the method of Bradford (Bradford, 1976). It should be noted that this report belongs to a series of articles related to pesticides, including carbamates, biotransformations. Microsomal preparations, cytochrome P450 enzyme activity characterizations and incubations were performed within 12 months during 2007/2008. The initial screening and identification of the metabolites by LC-TOF and quantiﬁcations and fragmentations by LC/MS-MS were carried out over 2008/2009. Data analysis and interpretations as well as publications were performed over prolonged period.

- 1. **Incubation and metabolite identiﬁcation**

The standard incubation mixture contained 50 and 100 µM furathiocarb, 0.15 mg pooled liver microsomal protein (n = 10), and 1 mM NADPH in a ﬁnal volume of 200 µl of 0.1 M phosphate buffer (pH 7.4). Furathiocarb was prepared once a week in dimethylsulfoxide (DMSO; ﬁnal amount in the reaction medium 1.0%). After a 2-min incubation at +37 ◦C in a shaking incubator block, the reaction was started by adding NADPH. The mixture was incubated at +37 ◦C for 30 min and the reaction was stopped with 600 µl of ice-cold acetonitrile containing an internal standard. All incubations were carried out in triplicate.

To measure the main furathiocarb metabolites of recombinantly expressed CYP enzymes (rCYPs), the standard incubation mixture (200 µl) contained 0.1 M phosphate buffer (pH 7.4), 1 mM NADPH, 100 µM benfuracarb, and rCYPs (50 pmol CYP per ml). Incubations were carried out according to the manufacturer’s instructions. Shortly, the reaction was started by adding recombinant enzymes to the preincubated eaction mixture (2 min at +37 ◦C), mixed gently and incubated for 30 min at +37 ◦C in an incubator block without agitation. Otherwise, the incubation protocol and analytical method were similar to those for microsomal incubations.

Samples were centrifuged before analysis for 10 min at 10000 × g. Chromatographic separation was carried out with the Waters Alliance 2690 HPLC system (Waters Corp., Milford, MA). The column used was a Waters Atlantis T3 (2.1 mm × 100 mm, particle size of 3 µm) together with a Phenomenex C18 2.0 mm × 4.0 mm precolumn (Phenomenex, Torrance, CA). The temperature of the column oven was 45 ◦C. The eluent ﬂow rate was 0.4 mL/min. The eluents used were ultrapure-grade water containing 0.1% acetic acid (A) and methanol (B). A linear gradient elution from 5% B to 75% B in 8 min was applied. Solvent B was thus maintained at 98% for 3 min before re-equilibration (6 min). The total analysis time was 17 min.

- 1. **Mass spectrometry**

The initial screening of the present and accurate mass measurements of compounds were carried out using a Micromass LCT (Micromass, Altrincham, UK) time of flight (TOF) mass spectrometer equipped with a Z-Spray ionization source. A generic positive electrospray ionization method was used for all substrates and metabolites. The capillary voltage was 4000 V, cone voltage 23 V, and desolvation and source temperatures 300 and 150°C, respectively. Nitrogen was used as the desolvation and cone gas with flow rates of 780 and 300 L/h. The mass spectrometer and HPLC system were operated under Micromass MassLynx 3.4 software. For exact mass measurements of metabolites in carbofuran metabolic pathways, the lock mass was *N*-1-naphthylphthalimide ([M+H]^+^ at *m/z* 274.0868), and it was delivered into the ionization source through a T-union using a syringe pump (Harvard Apparatus, Holliston, MA).

The quantification (multiple reaction monitoring, MRM) and fragmentation measurements were performed with a Micromass Quattro II triple quadrupole instrument equipped with a Z-spray ionization source. The capillary voltage was 4000 V, and desolvation and source temperatures 280 and 150°C, respectively. The collision gas was argon with a CID gas cell pressure of 2.0 × 10^3^ mbar. Nitrogen was used as the drying and nebulizing gas with flow rates of 450 and 15 L/h. The selected reaction monitoring (SRM), collision energies, and sample cone voltages for metabolites are presented in the study Figure 1. External standards were measured in the beginning, middle, and end of the experiment to ensure the quality of the analysis. Intraday coefﬁcients of variation were less than 20% throughout the quantitation range of 2.5–300 µM.

- 1. **The identification and quantification of furathiocarb metabolites**

Analytical standards were available for furathiocarb, carbofuran, 3-hydroxycarbofuran, 3-ketocarbofuran, 3-keto-7-phenolcarbofuran, 3-hydroxy-7-phenolcarbofuran, and 7-phenolcarbofuran. The identification was based on the comparison of exact masses and fragmentation patterns of the authentic standards with peaks in the incubates. Quantification was also based on the peak heights of the authentic standards.

Because analytical standards were not available for the presumed furathiocarb hydroxylated and sulfoxidated metabolites, they were tentatively identified on the basis of exact masses and fragmentation patterns and they were quantitated by using the calibration curve of furathiocarb, assuming their responses to be approximately equal. The lower limit of quantitation was 0.5 μM for all compounds. External standards were measured in the beginning, middle, and end of the experiment to ensure the quality of the analysis. Intraday coefficients of variation were less than 20 % throughout the quantitation range of 2.5 –300 μM.

- 1. **Incubation and analysis conditions for model P450 substrate activities in vitro**

Metabolites of bupropion, amodiaquine, tolbutamide, omeprazole, dextromethorphan, chlorzoxazone, and midazolam were analyzed by a Shimadzu VP series high-performance liquid chromatograph with an autoinjector (Shimadzu, Kyoto, Japan). The analytical column was a Waters Symmetry C_18_ (3.9 mm x 150 mm, particle size of 5 µm) together with a Lichospher 100 RP-18 4.0 mm x 4.0 mm precolumn (Merck, Darmstadt, Germany). Chromatographic methods were isocratic, except in the case of omeprazole when a linear gradient elution from 15 to 35% A in 8 min was used. Mobile phases were pumped at a flow rate of 1.0 ml/min. The injection volume used was 20 µl. The concentrations of metabolites were calculated from peak height ratios of the UV chromatograms on the basis of standard calibration curves of authentic metabolites. Metabolites of ethoxyresorufin, ethoxycoumarin, coumarin, and pentoxyresorufin were analyzed fluorometrically. All of the incubation and analysis conditions are summarized in Table 2. Furathiocarb was added in two concentrations (50 and 150 µM) to the incubation mixture with a small volume of DMSO as solvent. Fresh diuron dilutions from stock solution of DMSO were used for each assay. The final amount of DMSO was 1% in incubation mixtures. For chlorzoxazone 6-hydroxylation the solvent was evaporated because of the potent inhibition effect of DMSO on CYP2E1, and the residue was diluted with 10 µl of lipid solution. Lipid solution was prepared by adding 8 µl of dimethyldioctadecylammonium-bromide in chloroform (100 mg/ml) to 200 µl L-α-phosphatidylethanolamine dioleyl [C18:1 (cis)-9] in chloroform. This mixture was evaporated with a stream of nitrogen flow, 2 ml of water was added, and the final mixture was sonicated (Branson Sonifier 250 GWB) on an ice bath for 10 min by using 50% power. CYP-mediated activities in a panel of human hepatic microsomes (*n* = 10) are summarized in Table 3.

- 1. **Correlation with Model P450 Substrate Activities.**

Cytochrome P450 enzyme activity characterizations as well as incubations with individual human hepatic microsomes were performed at the same time. A bank of 10 livers was used to assess the metabolism of furathiocarb in individual livers as well as to correlate the activities with model P450 substrate activities. A correlation was performed between the formation of furathiocarb metabolites and each CYP activity across the human liver set. For all data points the mean of duplicate incubations were used. For all data points the means of duplicate incubations were used. Bivariate linear Pearson’s correlation coefficients (r) were calculated between metabolite formations and model activities in livers. Quantification of furathiocarb biotransformation *in vitro* by ten individual human liver microsomes was performed with 50μM furathiocarb for carbofuran metabolic pathway and 150μM furathiocarb for furathiocarb metabolite A and B and a 20-min incubation time. Metabolites A and B concentrations at furathiocarb 50 μM concentration did not permit correlation studies.

- 1. **Human CYP-furathiocarb inhibitory interactions by N-in-one assay incubation** **using pooled human hepatic microsomes**

N-in-one assay was performed to investigate human CYP-furathiocarb inhibitory interactions. Furathiocarb (ﬁnal concentrations in the incubation mixture were 1, 5, 25, 50, and 100 µM) was added to the incubation mixture.

Bupropion and hydroxybupropion were a generous donation from GlaxoSmithKline (Research Triangle, NC, USA); midazolam and 1- hydroxymidazolam were obtained from F. Hoffmann-La Roche (Basle, Switzerland); and omeprazole, omeprazole sulphone, and 5-hydroxyomeprazole were obtained from AstraZeneca (Mölndal, Sweden). The metabolite standards dextrorphan, desethylamodiaquine, 6-hydroxychlorzoxazone, and hydroxytolbutamide were purchased from BD Biosciences Discovery Labware (Bedford, MA, USA). Formic acid and LichroSol GG acetonitrile were obtained from Merck KGaA (Darmstadt, Germany). All other chemicals were from Sigma Chemical Co. (St. Louis, MO, USA) and were of the highest purity available. Water was freshly prepared in-house with simplicity 185 equipment and was UP grade (ultra pure, 18.2 MX).

The N-in-one assay, analytical method and authentic standards have been described previously (Abass and Pelkonen 2013; Tolonen et al. 2007; Turpeinen et al. 2005). In brief, incubations contained 0.5 mg pooled microsomal protein (n = 10), 100 mM phosphate buffer (pH 7.4), 1 mM b-nicotinamide adenine dinucleotide phosphate, reduced form (NADPH), and all ten probe substrates. Substrates and their ﬁnal concentrations for the incubations were: melatonin (4 µM), coumarin (2 µM), bupropion (1 µM), amodiaquine (2 µM), tolbutamide (4 µM), omeprazole (2 µM), dextromethorphan (0.2 µM), chlorzoxazone (6 µM), midazolam (0.4 µM), and testosterone (1 µM). 200 µl acetonitrile containing phenacetin (0.5 µM) as an internal standard was used to stop the reaction after 20 min incubation at 37 °C. For analysis, the incubations were thawed at room temperature, shaken, and centrifuged for 10 min at 10000 x g. The supernatants were transferred to a Waters Total Recovery vial (Waters Corporation, Milford, MA, USA) for LC/MS–MS analysis, as previously modiﬁed and described (Tolonen et al. 2009).

**Table 1. Characterization of the human liver samples.**

| Liver | Age | Sex | Cause of death | Drug history | Liver pathology |
| --- | --- | --- | --- | --- | --- |
| HL20 | 54 | M | ICH | Diazepam^a^ | None |
| HL21 | 44 | M | ICH | Phenytoin^a^, alcohol abuse | Cirrhotic |
| HL22 | 40 | F | ICH | Dexamethasone^a^, nizatidine^a^, phenytoin^a^ | None |
| HL23 | 43 | M | ICH | Diazepam^a^, smoker | None |
| HL24 | 47 | M | ICH | No medication, smoker | None |
| HL28 | 21 | M | Stroke | Dexamethasone^a^, smoker | None |
| HL29 | 39 | F | ICH, SAH | Dexamethasone^a^ | None |
| HL30 | 53 | F | ICH, SAH | No medication | Steatosis |
| HL31 | 44 | F | ICH, SAH | No medication | Steatosis |
| HL32 | 62 | M | ICH, SAH | Metformin, alcohol abuse, smoker | None |

^a^ Drugs were administrated only during the last 24 hours before death; M, male; F, female; ICH, intracerebral hemorrhage; SAH, subarachnoidal hemorrhage; SDH, subdural haematoma.

**Table 2: Incubation and analysis conditions for model P450 substrate activities in vitro**

| **P450** | ***Reaction*** | **Conc. (μM)** | **Protein mg/ml** | **Cofactor System** | **Incubation Time min** | **Determination, λ nm** | **Eluent** |
| --- | --- | --- | --- | --- | --- | --- | --- |
| CYP1A1/2 | 7-Ethoxyresorufin-O-deethylation (EROD) | 1 | 0.1 | NADPH-regenerating | 5 | Fluorometric, 530/585 |  |
| CYP2A6 | Coumarin-7-hydroxylation (OH-COU) | 10 | 0.2 | NADPH-regenerating | 10 | Fluorometric, 365/454 |  |
| CYP2B6 | Bupropion hydroxylation (OH-BUP) | 50 | 0.4 | NADPH | 15 | UV-HPLC, 214/204 | 75% 50 mM *o*-phosphoric acid-buffer: 25% acetonitrile |
| CYP2C8 | Amodiaquine-desethylation (deEt-AMO) | 30 | 0.5 | NADPH | 20 | UV-HPLC, 342 | 70% 50 mM *o*-phosphoric acid-buffer: 30% acetonitrile |
| CYP2C9 | Tolbutamide hydroxylation (OH-TOL) | 200 | 0.15 | NADPH-regenerating | 20 | UV-HPLC, 236/204 | 70% 50 mM o-phosphoric acid-buffer: 30% acetonitrile |
| CYP2C19 | Omeprazole-5- hydroxylation (5-OH-OME) | 40 | 0.1 | NADPH | 20 | UV-HPLC, 304/204 | 25 mM o-phosphoric acid-buffer (A) and acetonitrile (B) |
| CYP2D6 | Dextromethorphan-*O*- desmethylation (*O*-dem-DEX) | 10 | 0.1 | NADPH-regenerating | 20 | UV-HPLC, 204/280 | 75% 50 mM o-phosphoric acid-buffer: 25% acetonitrile |
| CYP2E1 | Chlorzoxazone-6-hydroxylation (OH-CLZ) | 100 | 0.1 | NADPH-regenerating | 20 | UV-HPLC, 282/204 | 70% 50 mM o-phosphoric acid-buffer: 30% acetonitrile |
| CYP3A4 | Midazolam-1'-hydroxylation (1-OH-MDZ) | 10 | 0.1 | NADPH-regenerating | 5 | UV-HPLC, 245/204 | 60% water: 40% acetonitrile |
| CYP3A4 | Omeprazole sulfoxidation (SO2-OME) | 40 | 0.1 | NADPH | 20 | UV-HPLC, 304/204 | 25 mM o-phosphoric acid-buffer (A) and acetonitrile (B) |

**Table 3: CYP-mediated activities in a panel of human hepatic microsomes (n = 10).**

| Enzyme | Reaction |  | CYP activities measured in pooled human hepatic microsomes  (pmol/(mg protein*min) | |
| --- | --- | --- | --- | --- |
|  |  |  | Range of values | mean ± SD |
| CYP1A1/2 | 7-Ethoxyresorufin-O-deethylation (EROD) | | 3.8 – 109.8 | 50.0 ± 31.7 |
| CYP2A6 | Coumarin-7-hydroxylation (OH-COU) | | 95.8 – 549.2 | 281.6 ± 167.3 |
| CYP2B6 | Bupropion hydroxylation (OH-BUP) | | 4.5 – 220.5 | 73.1 ± 61.9 |
| CYP2C8 | Amodiaquine-desethylation (deEt-AMO) | | 152.6 – 362.4 | 238.8 ± 65.4 |
| CYP2C9 | Tolbutamide hydroxylation (OH-TOL) | | 79.8 – 269.3 | 148.6 ± 55.9 |
| CYP2C19 | Omeprazole-5- hydroxylation (5-OH-OME) | | 21.5 – 124.1 | 55.8 ± 35.8 |
| CYP2D6 | Dextromethorphan-*O*- desmethylation (*O*-dem-DEX) | | 67.9 – 229.6 | 156.9 ± 58.9 |
| CYP2E1 | Chlorzoxazone-6-hydroxylation (OH-CLZ) | | 136.0 – 773.5 | 397.4 ± 202.2 |
| CYP3A4 | Midazolam-1'-hydroxylation (1-OH-MDZ) | | 511.6 – 4568.2 | 1499 ± 1336 |
| CYP3A4 | Omeprazole sulfoxidation (SO2-OME) | | 12.9 – 130.5 | 49.6 ± 40.8 |

**References**

Abass, K. and Pelkonen, O. The inhibition of major human hepatic cytochrome P450 enzymes by 18 pesticides: Comparison of the N-in-one and single substrate approaches. Toxicology in Vitro 27:1584-8; 2013.

Tolonen, A., Turpeinen, M., Pelkonen, O. Liquid chromatography–mass spectrometry in in vitro drug metabolite screening. Drug Discov Today 14:120-33; 2009.

Tolonen, A., Petsalo, A., Turpeinen, M., Uusitalo, J., Pelkonen, O. In vitro interaction cocktail assay for nine major cytochrome P450 enzymes with 13 probe reactions and a single LC/MSMS run: analytical validation and testing with monoclonal anti-CYP antibodies. J Mass Spec 42:960-6; 2007.

Turpeinen, M., Uusitalo, J., Jalonen, J., Pelkonen, O. Multiple P450 substrates in a single run: rapid and comprehensive in vitro interaction assay. Eur J Pharm Sci 24:123-32; 2005.
